# Impact of a New Virtual Health Humanities Approach for Collaborative Online International Learning: Mixed Methods Study

**DOI:** 10.2196/84474

**Published:** 2026-10-28

**Authors:** Heather Meredith Gaunt, Kwang Meng Cham, Florian Knothe, Chee Han Lim, Rosie Shea, Hui Ting Chng

**Affiliations:** 1 Museums and Collections Department, Chancellery, University of Melbourne, Grattan Street, Parkville, 3010, Australia, 61 0447068084, 61 3 9347 5904; 2 Department of Optometry and Vision Sciences, Faculty of Medicine, Dentistry and Health Sciences, University of Melbourne, Melbourne, Australia; 3 University Museum and Art Gallery, School of Humanities, Faculty of Arts, University of Hong Kong, Hong Kong, China; 4 NUS College, National University of Singapore, Singapore, Singapore; 5 Department of Medical Education, Faculty of Medicine, Dentistry and Health Sciences, University of Melbourne, Melbourne, Australia; 6 Department of Pharmacy and Pharmaceutical Sciences, Faculty of Science, National University of Singapore, Singapore, Singapore

**Keywords:** internationalization, intercultural learning, intercultural perspectives, e-learning, collaborative online international learning, COIL, global classroom, health humanities, medical humanities, object-based learning, OBL, museum-based health education, MB-HE, cultural humility, cultural diversity, mixed methods

## Abstract

**Background:**

This study presents a novel virtual health-humanities approach to collaborative online international learning, developed and delivered between 2023 and 2025. The approach, which we describe for the purposes of this study as a “Health Humanities Global Classroom” (HHGC), aimed to raise health professions students’ awareness of intercultural perspectives, potentially fostering more interculturally attuned health professionals. In the HHGC, students across 3 international universities participated synchronously in a virtual object-based learning seminar using shared teaching expertise and digitized artworks and museum objects drawn from the universities’ diverse art and cultural collections.

**Objective:**

This mixed methods research study aimed to assess the HHGC’s impact on encouraging a deeper awareness of intercultural perspectives in participants.

**Methods:**

A mixed methods study was conducted, involving 58 health professions students from universities in Australia, Singapore, and Hong Kong in February 2025 and March 2025. All students participated in a 2-hour online seminar with the theme of global health and well-being, during which they undertook guided exploration of virtual museum objects and artworks. Data on students’ seminar experiences were collected through presurveys and postsurveys, written reflections created during the seminar, and online focus groups conducted immediately afterward.

**Results:**

All 58 students participated in the focus groups. Among the participants, 84% (49/58) completed the postseminar survey; 96% (47/49) agreed that the seminar increased their awareness of how they communicate and exchange meanings with peers from diverse cultures; 98% (48/49) agreed that engaging with the museum objects in the seminar with peers heightened their awareness of diverse perspectives; and 86% (42/49) agreed that the seminar increased their awareness of local health in interprofessional contexts. Four primary themes were identified through qualitative analysis, including (1) key learning outcomes (intercultural, analytical, and empathic capacities, and knowledge acquired), (2) enabling factors (pedagogy, environment, and peer interaction), (3) barriers to learning (pedagogical approach, learning resources, time factors, and virtual constraints), and (4) novel and distinctive experiences (international, arts-integrated learning).

**Conclusions:**

This study provides early evidence for an innovative and accessible pedagogical approach. The HHGC is promising as a valuable educational activity, with positive student engagement in a safe environment for intercultural dialogue mediated by virtual museum objects and artworks. This approach may be useful to enhance participants’ awareness of cultural humility.

## Introduction

### Background

Providing culturally sensitive and appropriate care is a cornerstone of modern global health equity. Medical and educational institutions increasingly seek curricula that foster intercultural competence [1-6]. While there is currently no consensus on the definition of “intercultural competence,” it is widely understood as the “ability to communicate effectively and appropriately in intercultural situations based on one’s knowledge, skills, and attitudes” [7,8]. Additionally, as a pedagogical objective, the acquisition of “intercultural competence” betrays an assumption that culture is a finite body of knowledge to be mastered, a view that risks reducing complex human worlds to a static checklist of traits [7].

A combination of methods such as lectures with simulations, role-playing, and community engagement with diverse populations has been found to enhance intercultural competence [9]. Experiential learning, such as international placements, has also made a positive impact on the development of cultural competence among health professional students [10]. However, these incur financial costs and logistical barriers, making them inaccessible for many. The reach of such interventions is further limited by packed, discipline-specific health curricula that relegate humanistic approaches that cultivate essential skills like empathy, observation, and comfort with ambiguity to their periphery. This creates a critical gap: a need for scalable, accessible, and pedagogically innovative methods to mainstream intercultural competence learning.

To address these pedagogical challenges, we designed a new humanities-based methodology which could be used as an intervention in an existing health professions curriculum. Our approach, which we describe as a “Health Humanities Global Classroom” (HHGC), builds on the known potential of 3 pedagogical approaches, specifically museum-based health education (MB-HE), object-based learning (OBL), and collaborative online international learning (COIL).

### MB-HE

MB-HE uses carefully facilitated engagement with art and museum collections to support the development of transferable skills and ways of thinking among health professionals, such as visual observation, tolerance for ambiguity, awareness of bias and diversity of perspectives, empathy, and self-reflection. By connecting these experiences in the museum to clinical practice, the approach aims to promote more humanistic, patient-centered care, and simultaneously support well-being and reduce burnout [11,12]. MB-HE activities are commonplace in many universities’ health education curricula around the world and are extensively reported as an effective pedagogical approach [11,12]. Particularly since the COVID-19 pandemic, this approach has been translated into online learning environments with varied success [13-15]. Online collaborative intercultural peer group activity in the global health educational context has been beneficial for student learning by complementing existing teaching in global health [16].

### OBL

OBL is a student-centered, experiential pedagogy that uses direct engagement with artworks, museum objects, and artifacts, and other types of material culture such as archives or scientific instruments, to stimulate observation, enquiry, and knowledge construction [17]. Differing from MB-HE, it is applied in any disciplinary context and, in its simplest form, is taught as a small-group “hands-on” investigation of a single object, where meaning or interpretation is built through discussion [17]. OBL has been adopted globally and is increasingly recognized as a powerful tool to promote ethical considerations of material culture, collections, and power [18].

### COIL

COIL is an internationalized educational approach in which educators from different countries and universities co-design and cofacilitate curriculum-integrated learning experiences that bring students together through real-time online interaction and collaborative inquiry. By engaging in sustained intercultural dialogue and joint academic tasks over the course of a “project” or part of a curriculum, students can potentially develop intercultural competence and understandings of global perspectives, as well as build collaborative and teamwork skills [19,20]. As Hackett et al [21] noted, measuring whether COIL programming successfully improves participants’ intercultural competence is difficult, so further investigation is required to clarify its effectiveness in this area [21].

### Objectives

Building on the aforementioned pedagogies, we hypothesized that a teaching and learning approach that privileges awareness of multiple perspectives on a relevant health topic across a global context, using OBL with diverse cultural artifacts as mediating objects, in a virtual COIL context, would allow students to develop a deeper awareness of aspects of intercultural competence. We shaped our HHGC methodology to create a “third space” for intercultural dialogue [22], where learning is a situated activity and where knowledge is coconstructed within a temporary “community of practice” formed by the international student group [23]. We speculated that, through the OBL approach, the shared (virtual) artwork or cultural artifact would function as a neutral ground upon which students could negotiate complex meanings and explore sensitive topics without feeling personally exposed. The approach would thus foster a mode of inquiry that was analytical and creatively engaged. We undertook a research study that aimed to give us a deeper understanding of participants’ experiences of our HHGC methodology, and the potential of the methodology to support students’ development of intercultural competence.

## Methods

### Overview

The development phase of our HHGC methodology has been published previously in brief [24]. In this article, we report the results of a pilot delivery of the HHGC methodology conducted in early 2025 with health students from 3 international universities: the University of Melbourne (UoM), the University of Hong Kong (HKU), and the National University of Singapore (NUS). The full HHGC project, including the learning components and associated research to understand its impact, is shown in Figure 1, and described in detail below.

The identical HHGC seminar was delivered in English and ran for 2 hours. The seminar was delivered on 6 occasions, each attended by between 8 and 11 participants, and each participant took part in a single session. To maximize the cultural diversity of the learning experience, we allocated students as evenly as possible from each university site, according to student availability. The mix of students in each seminar is shown below (Table 1).

**Figure 1.**
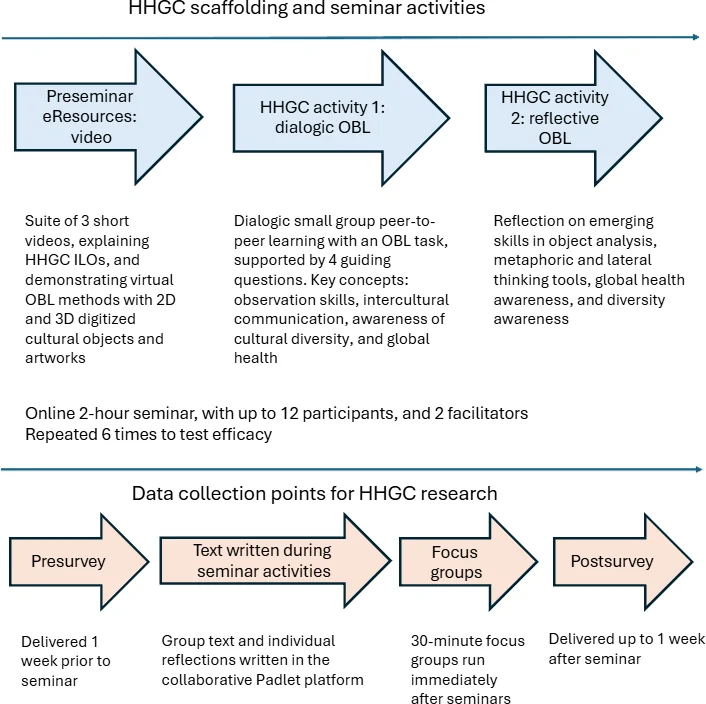
Health Humanities Global Classroom (HHGC) program components. ILO: intended learning outcome; OBL: object-based learning.

**Table 1.** Distribution of participants across 6 Health Humanities Global Classroom (HHGC) seminars (by university).

Seminar repeat delivery, n	Student from UoM^a^, n	Student from HKU^b^, n	Student from NUS^c^, n	Total seminar participants, n
1	4	1	5	10
2	4	1	3	8
3	3	1	5	9
4	3	2	6	11
5	2	1	6	9
6	5	2	4	11
Total participants across all seminars:				58

a UoM: University of Melbourne.

b HKU: University of Hong Kong.

c NUS: National University of Singapore.

The seminars were facilitated by 2 members of the team, one with health professional expertise, and one with museum expertise, representing 2 different universities. Across the 6 occasions, all research team members contributed to facilitation at least once. Facilitators closely followed a script cocreated by all members of the team to ensure uniformity of the participants’ experience. The seminar theme was broad, with discussions shaped around thoughtful comparisons of health and well-being in the contexts of each university’s local and national environment. Using Anderson and Krathwohl’s revision of Bloom’s Taxonomy [25], our intended learning outcomes for the pilot HHGC (encompassing Levels 2 to 6 of the Taxonomy) were as follows: (1) use effective communication with students from different disciplinary backgrounds and cultures (Level 2: Understand and Level 3: Apply); (2) apply effective teamwork skills with students from different disciplinary backgrounds and cultures (Level 3: Apply and Level 4: Analyze); (3) develop an understanding of different cultural and global perspectives on health care (Level 2: Understand and Level 4: Analyze); and (4) build observational, creative, and critical thinking skills to discuss the presented artwork or museum object (Level 4: Analyze, Level 5: Evaluate, and Level 6: Create).

The OBL content for discussion consisted of a virtual group (e-Gallery) of 24 artworks and museum objects, which we selected in equal numbers from the cultural collections of the 3 universities (Figure 2).

Artworks were selected by the museum professionals on the team, who worked closely together online to curate the final group to provide a balance of cultural and historical contexts of the artworks’ production and a rich potential to trigger discussions about the human body, health, or well-being.

Facilitators formed breakout-room teams of 3 to 4 participants at the start of each seminar, aiming, where possible, for each team to include at least 1 student from each university site. In the facilitated OBL activities, teams selected 1 object from the e-Gallery for in-depth exploration and discussion. Four guiding questions scaffolded the team discussions, encouraging students to move logically through a process: to undertake close observation of the object; to build a detailed, collective description; to consider and share their emotional responses; and to reflect on how their cultural perspectives might shape their observations. Teams documented their close observations on the collaborative online platform Padlet, including how they connected them to the HHGC’s overarching theme of health [26]. In the final part of the seminar, students each selected a new object and crafted short reflective statements that captured their understanding of their object, and how the group discussion may have influenced or changed their analysis. Participants were encouraged throughout the seminar to use an enquiring approach, to make direct or lateral connections between the artworks and the broad theme of global health, and to contribute these actively to the group discussion.

**Figure 2.**
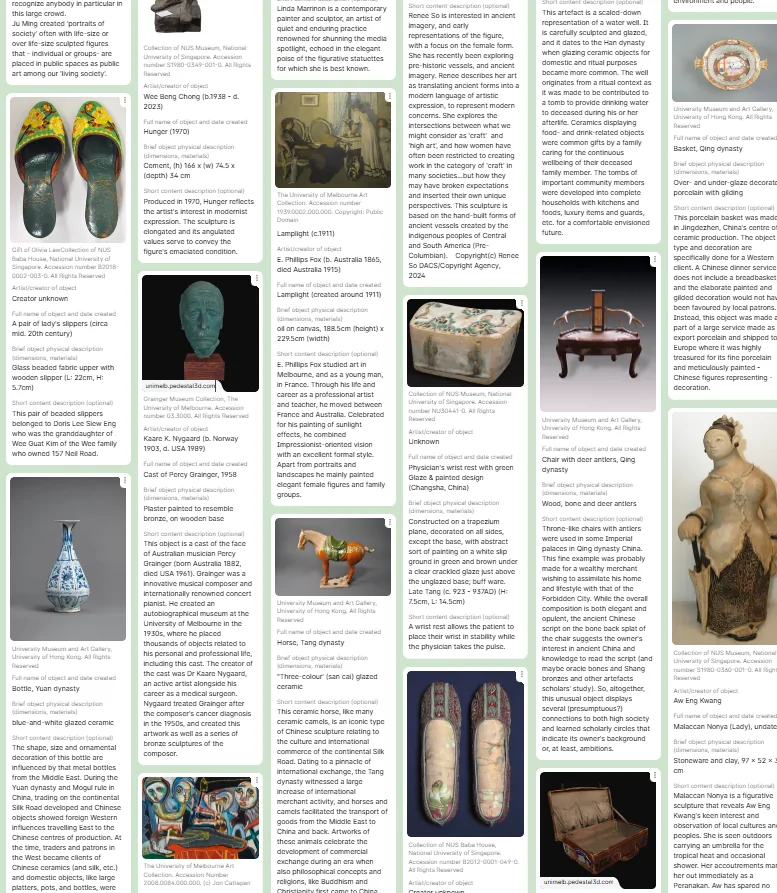
Screen capture of a section of the e-Gallery resource of cultural objects and artworks.

### Ethical Considerations

We designed a mixed methods research study to evaluate the impact of the HHGC on intercultural learning for participants. Ethical approval has been granted by all 3 universities where students were participating in this project: The University of Melbourne Office of Research Ethics and Integrity (reference: 2024-28502-54028-3; approval date: May 31, 2024), National University of Singapore Institutional Review Board (reference: NUS-IRB-2024-467; approval date: September 27, 2024), and The University of Hong Kong Research Ethics Institutional Review Board (protocol: UW 24-583; approval date: October 25, 2024). All participants provided informed consent through Qualtrics, and participation was entirely voluntary. All surveys were anonymous, and participants were not identifiable. All data from focus group recordings and text data created by participants during the HHGC activities were anonymized and deidentified to safeguard participant information. Participants were reimbursed for their time with a US $65 gift voucher.

### Participants

Participants in the HHGC were diverse, drawn from students studying at different levels and in different curriculum contexts at the 3 universities. A convenient sampling approach was undertaken to recruit participants at each of the university sites, via a recruitment email posted on the learning management system from members of the research team to specific cohorts (for example, students studying an elective “Medical Humanities” topic at UoM). In the presurvey, delivered to participants via Qualtrics up to 2 weeks prior to the scheduled seminar, we sought to understand the participants’ demographic and contextual (social, cultural, and historical) details, to learn more about their previous experiences in online learning and museum-based learning, and their current perception of confidence relating to intercultural communication ability (Table 2).

**Table 2.** Presurvey questions, Health Humanities Global Classroom.

Question number	Question	Response selection
1	Which university are you currently studying at?	University of Melbourne, University of Hong Kong, and National University of Singapore
2	Which of the following area(s) of health is the focus of your current tertiary study? Select one or more answers.	Medicine, nursing, pharmacy, optometry, dentistry, audiology, social work, and other
3	At what year level of your degree are you currently studying?	Years 1, 2, 3, 4, 5, and 6
4	Have you participated in an online international learning experience previously?	Yes/no
5	If you answered yes to the question above, briefly describe that experience.	Free-text answer
6	Have you participated in a museum-based learning experience as part of your tertiary education?	Yes/no
7	If you answered yes to the question above, briefly describe that experience.	Free-text answer
8	What language/s do you speak at home?	Free-text answer
9	Rate your response to the following question: “I am confident in my ability to communicate and exchange meanings with colleagues from diverse cultures in a variety of contexts.”	Extremely confident, very confident, somewhat confident, not very confident, and not at all confident

Focus groups of approximately 30 minutes were conducted and recorded on Zoom following each seminar. We chose focus groups so that participants could build on each other’s ideas in a collaborative context, continuing the open and safe environment for sharing that had already been created in the seminar [27]. Focus groups were facilitated by members of the research team who had not taught in the preceding seminar; each facilitator followed a script with ethics-approved questions and prompts (Textbox 1). Each focus group had 3 to 6 participants, depending on the total number attending the seminar; the focus groups ran simultaneously in new, separate Zoom meetings beginning ten minutes after the seminar concluded. Focus-group participants were predetermined during the allocation of seminar attendees, with the aim of including as even a distribution from each university as possible. During the focus groups, participants were asked questions designed to explore how they individually experienced the seminar’s contextual factors. Focus groups’ transcriptions were anonymized, and each participant was given a code number.

Textbox 1. **The presurvey questions delivered to students who indicated interest in participating in the health humanities global classroom seminar.**

- What was your experience of participating in this seminar with international peers?
- What was the experience of exploring the global health theme in this specific seminar context like for you?
- What was your experience of sharing your personal perspectives and observations during the seminar?
- What was your experience of hearing the perspectives of the other students during the seminar?
- What was the experience of working with virtual museum objects like for you?
- Has this seminar been a unique experience for you in any way?

### Analysis

As noted in Figure 1, we collected quantitative data at the beginning and end of the data-collection phase using surveys, and qualitative data midstudy through in-seminar reflective texts on Padlet, and postseminar focus groups. Our surveys contained a mix of qualitative and quantitative items. We analyzed quantitative survey data in Qualtrics, reporting percentages for categorical variables and means for Likert-scale items. GraphPad Prism 6.07 (GraphPad Software Inc) was used for graphical presentation of the data. We reported only descriptive statistics (percentages and means) because this pilot study was an exploratory evaluation of a novel pedagogical intervention, with a modest convenience sample and immediate postsession measurement. In this paper, we did not include data or analysis from the in-seminar reflective texts; that material will be included in a future paper that focuses on the impact of our virtual OBL approach, addressing the technical challenges and opportunities of the pedagogy. Qualitative data from the focus groups were analyzed thematically to categorize the major themes, using the qualitative data analysis software NVivo (version 15; Lumivero). We used a qualitative data analysis approach described by Fereday and Muir-Cochrane for text-based data [28]. An initial codebook was developed by HMG. Coding was legitimated when RS, HTC, and KMC (representing 2 of the 3 participating sites) each independently checked the coding on 4 focus-group transcripts. The platform Spark AI (UoM) was used by HMG to run queries across the qualitative data set of focus groups to explore potential additional subcodes. Themes and subthemes derived from the codes were discussed by the team via email and Zoom meetings. Any disagreements were resolved by HMG. The final consensus was shared with the full team as a report produced in NVivo, listing themes, related codes, theme descriptions, and statistics on the number of files and references for each.

Finally, we adopted a convergent mixed methods approach in which descriptive quantitative measures of participants’ perceptions are interpreted alongside thematic qualitative data to provide complementary perspectives. Quantitative percentages describe the prevalence of positive perceptions across the cohort, while qualitative data explain how and why participants experienced these outcomes. In reporting, we therefore emphasized convergence (where high percentages aligned with recurrent qualitative themes) and complementarity (where qualitative data provided more nuance to the descriptive results).

All members of the research team were conscious of and reflected on their potential biases, recognizing that their diverse backgrounds as doctors, pharmacists, anthropologists, optometrists, clinical educators, museum curators, and educators may influence the interpretation of the results to some degree. Reflexive notes throughout the development and delivery of the pilot methodology and multiple discussions across the data analysis period ensured that coding decisions were anchored in the transcript data rather than researchers’ *a priori* expectations, as much as possible.

## Results

### Participant Demographics

A total of 67 students were recruited for this study and completed the preseminar survey in the week prior to the seminars. Of the 67 students, 58 attended an HHGC seminar, and the 9 who did not attend were either unable to find a suitable seminar time or faced unforeseen personal circumstances. Because of survey anonymity, we were unable to identify which presurvey respondents did not continue in the study. All 58 students who attended an HHGC seminar participated in the focus groups; 84% (49/58) of seminar attendees completed the postseminar survey. The 67 participants in the presurvey ranged across first to fourth years of their current degree across the 3 university sites, with a few having recently graduated (Tables 3 and 4).

**Table 3.** Presurvey responses from 67 participants indicating location and current year of studies.

University	First year; n	Second year; n	Third year; n	Fourth year; n	Recently graduated; n	Total responses; n
NUS^a^	3	17	7	6	0	33
HKU^b^	3	3	1	3	0	10
UoM^c^	0	1	15	4	4	24
Total	6	21	23	13	4	67

a NUS: National University of Singapore.

b HKU: University of Hong Kong.

c UoM: University of Melbourne.

**Table 4.** Presurvey responses from 67 participants showing university site, and their current disciplinary focus/foci.

Site	Medicine; n	Pharmacy; n	Nursing; n	Population and global health; n	Nursing, population and global health, and health and well-being; n	Pharmacy and health and well-being; n	Other, n	Total, n
NUS^a^	4	21	3	0	0	3	2	33
HKU^b^	3	0	1	2	2	0	2	10
UoM^c^	23	0	0	0	0	0	1	24
Total	30	21	4	2	2	3	5	67

a NUS: National University of Singapore.

b HKU: University of Hong Kong.

c UoM: University of Melbourne.

All participants were fluent in English, and a large proportion were multilingual, speaking other languages at home, including Sinhalese, Mandarin, French, and Italian. From the presurvey data, 18% (12/67) had previously participated in an online international learning experience; 45% (30/67) had participated in a museum-based learning experience as part of their tertiary education, including single sessions in local history, art, or anatomy museums, and in some cases, more extensive museum experiences through enrollment in medical humanities courses.

### Quantitative Analysis

From the 58 students, 84% (n=49) of seminar participants completed the postseminar survey (Figure 3). In this survey, 96% (47/49) of respondents agreed that the seminar had increased their awareness of how they communicated and exchanged meanings with peers from diverse cultures. Relating to health knowledge, 86% (42/49) agreed that the seminar had increased their awareness of local health in interprofessional contexts, while 92% (45/49) agreed that they had an increased understanding of health from a global perspective. Relating to the virtual OBL experience, 98% (48/49) agreed that engaging with the diverse museum objects was thought-provoking and that engaging with these objects in a peer learning environment heightened their awareness of diverse perspectives. The same proportion of participants also agreed that this COIL experience with international peers was an effective way to increase their awareness of intercultural competence.

**Figure 3.**
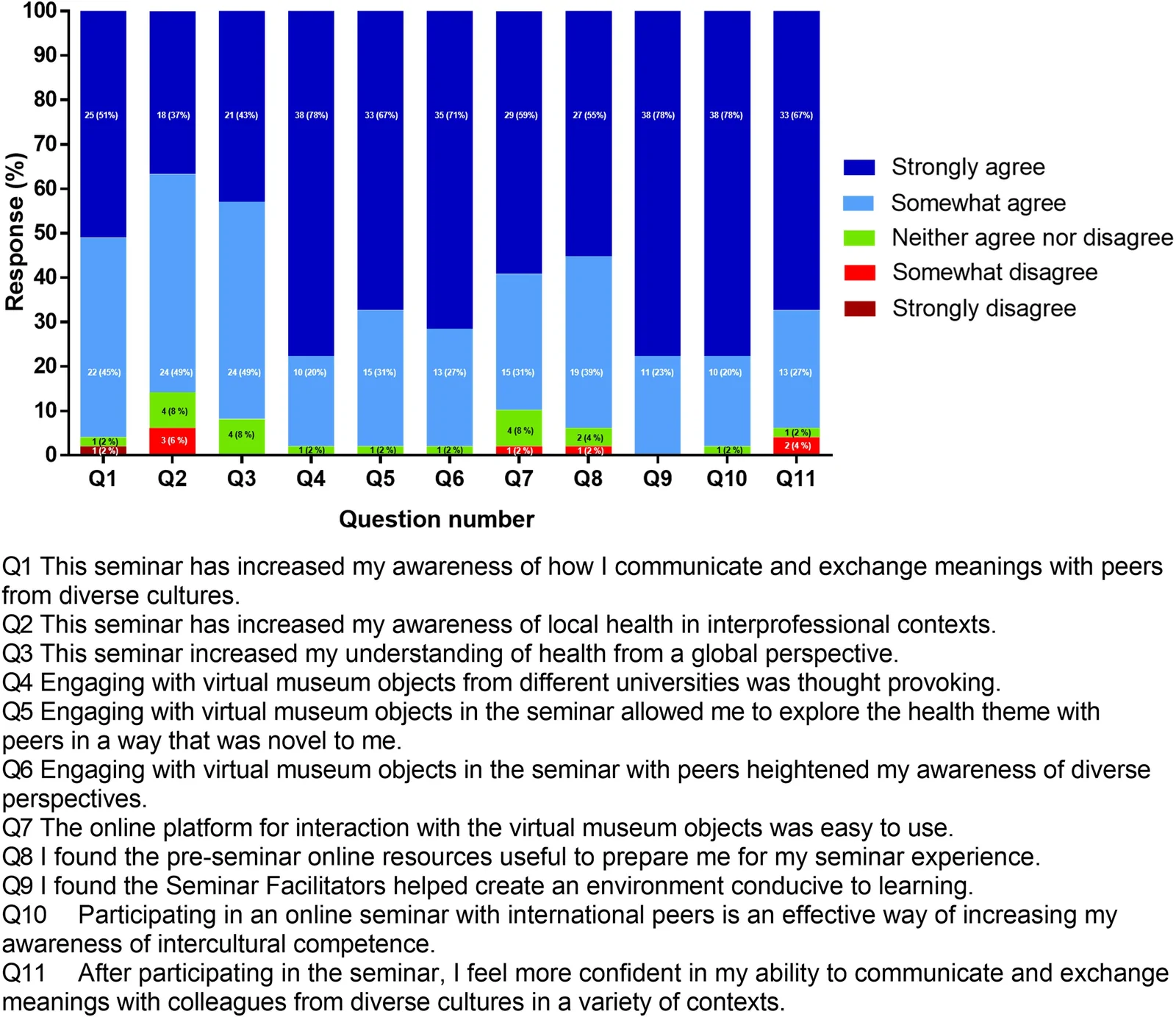
Participant responses to postsurvey questions.

In the presurvey and postsurvey, participants were asked to describe their immediate perception of confidence in communicating across cultures (Q9: presurvey; Q11: postsurvey). While our presurvey question asked about their current self-perception of confidence in communicating in intercultural contexts, the postsurvey question sought to identify any change in their perception of their confidence after seminar participation. Prior to the seminar, 92% of students who completed the survey were confident to some degree (“somewhat confident,” “very confident,” or “extremely confident”), with around half of that group sitting in the “somewhat confident” category. In the postsurvey, we saw that 94% (46/49) perceived an increase in their confidence through the experience (Figure 4).

**Figure 4.**
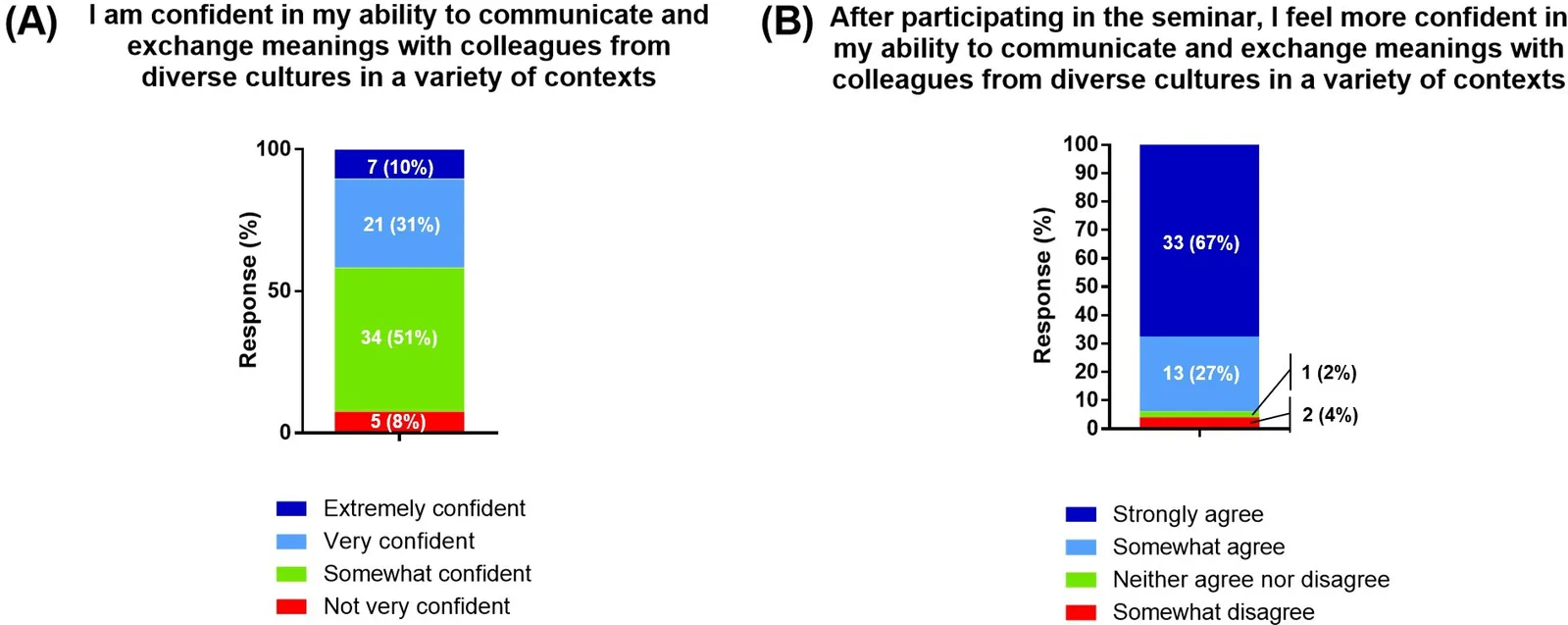
Side-by-side graphic comparisons of (A) preseminar and (B) postseminar survey questions that explored participants’ immediate self-perception of confidence in their intercultural communication ability.

### Thematic Analysis

Four key themes, with multiple subthemes, emerged from our thematic analysis of the textual data derived from transcriptions of the 12 focus groups and text-based responses in the postsurvey. The themes presented students’ perceptions of their experiences of the seminar across three distinct areas, namely (1) their key learning outcomes, the enabling factors which allowed them to engage and learn effectively; (2) the barriers to learning they encountered; and (3) their articulation of what was unique or novel about the seminar experience. These themes are summarized in Table 5. The themes were listed in the order of frequency with which they occurred in the data. We also identified a fourth theme, which brought together their suggestions for improvements to the seminar, or future use of the methodology in health education. We did not include this fourth theme in the primary findings, as we considered that the data should be considered separately from that which reported their experience in the HHGC seminar itself.

**Table 5.** Students’ experience of the Health Humanities Global Classroom (HHGC): qualitative themes and summary of subthemes.

Key themes	Subthemes
1. Key learning outcomes: intercultural, analytical, and empathic capacities, and knowledge acquired	Self-perception of intercultural capacities enhanced, including new understandings of the influence of cultural lenses, effectiveness of diverse perspectives, and specific capacities regarding communication across cultures. Self-perception of skills and capacities enhanced, including observation skills, analytic style, increased empathy awareness, and importance of emotions. Knowledge acquired in health and global health, and personalized care. Knowledge acquired about art or culture.
2. Enabling factors: pedagogy, environment, and peer interaction	Exposure to diverse perspectives including from international peers. Engaging e-resources, and application of OBL^a^ pedagogy. Safe, positive, comfortable, and inclusive learning environment. Collaborative peer discussions and exposure to peer expertise. Sense of commonality among peers. Using art to understand health Contribution of expert and supportive facilitators Encouragement of exploratory lateral thinking Small group size
3. Barriers to learning: pedagogical approach, learning resources, time factors, and virtual constraints	Limitations of e-resources as compared to experiencing physical artworks or objects. Unfamiliarity with the approach of using art to explore health. Challenge of virtual learning environment (as compared to a physical environment). Time constraints of a single seminar
4. Novel and distinctive experiences: international, art-integrated learning	Confluence of multiple HHGC factors (including virtual OBL with art, international peer exposure, and linked health theme, and so on). Difference of humanities learning environment compared with standard health education. Intersection of humanities and health Discussing art with international peers

a OBL: object-based learning.

### Theme 1: Key Learning Outcomes

In terms of learning outcomes, participants expressed the acquisition of analytical, intercultural, and empathic capacities, and specific knowledge. The capacity most frequently identified by participants was an enhancement of their intercultural awareness. The second most common capacities we grouped as personal skills, such as observational skills, and critical or analytic thinking.

### Theme 2: Enabling Factors

The second theme helped us to understand the enabling factors that contributed to participants’ learning. The highest-cited factor we identified was the students’ exposure to diverse perspectives (including those from international peers). Engagement with e-resources and the OBL pedagogy was the second-highest factor. A positive and inclusive learning environment was the third most common factor identified.

### Theme 3: Barriers to Learning

This theme allowed us to understand the barriers students reported as interfering with their learning or engagement. The highest-cited factor focused on various limitations of the e-resources, mostly in comparison to a face-to-face experience with physical artworks or objects. The second highest barrier was unfamiliarity with the lateral approach of using art to explore health. However, all responses that cited this barrier included descriptions of how they were able to improve their confidence with the lateral approach during the seminar: what was initially experienced as a difficulty became a positive through the influence of other factors, including more time to work on a skill, learning from their peers, or effective facilitator interactions. As 1 participant observed:

*For me, it was quite intimidating at first, because I didn't really have much experience with connecting humanities to health, and so I had to sort of come up with responses and opinions in such a short frame of time. … But once the flow, once the whole thing started rolling, and we each got more involved and then, it became smoother, the process of thinking and analyzing.*

[2.1P2]

It is important to note that what some participants perceived as barriers were not perceived as barriers by others. For example, the online (Zoom platform) learning experience was identified by some participants as a highly effective way of engaging synchronously with international peers, while others found it to be less optimal than face-to-face learning.

### Theme 4: Novel and Distinctive Experiences

The fourth theme focused on the uniqueness of the HHGC experience. Participants identified a range of factors that were novel to them, such as the opportunity to explore art in relation to health topics, accessing virtual galleries of artworks scanned in three dimensions, and synchronous learning with international peers from diverse backgrounds. The most highly cited aspect was the confluence of more than three HHGC factors. One participant described this as “the whole package” (6.1P4). We compare the quantitative and qualitative findings in Table 6.

**Table 6.** Side-by-side convergence of Health Humanities Global Classroom (HHGC) quantitative and qualitative data.

Analytic domain (theme)	Quantitative findings	Qualitative evidence (theme summary and representative quotes)
Key learning outcomes: intercultural, analytical, and empathic capacities, and knowledge acquired.	98% (48/49) agreed engaging with objects with peers heightened awareness of diverse perspectives. 98% (48/49) agreed the COIL^a^ experience was effective to increase awareness of intercultural competence. 92% (45/49) increased understanding of health from a global perspective. 86% (42/49) increased awareness of local health in interprofessional contexts.	“[I] understood much more clearly - through personal experience obtained from completing the exercises - how culture informs how we see the world and how layered and complicated that can be.” (PS.P7) “I feel like …we … can also understand different perspectives from our own very different cultural contexts, like, from our international peers. And that I feel like such new perspective will allow us to gain a very holistic overview, which actually is something I feel like is very needed… given our current global landscape.” (2.1P4) “I learned to link my thought processes on health-related concepts to the culture in which the item in question is produced, beyond simply making links to the item itself.” (PS.P22)
Enabling factors: pedagogy, environment, and peer interaction	100% (49/49) agreed that the seminar facilitators helped create an environment conducive to learning. 98% (48/49) agreed engaging with virtual museum objects was thought-provoking. 90% (44/49) agreed that the online platform for interaction with the virtual museum objects was easy to use. 94% (46/49) agreed that the preseminar online resources were useful preparation for the seminar experience.	“Engaging with peers from different linguistic and cultural backgrounds also strengthened cross-cultural communication skills and underlined the need for cultural sensitivity in global health work in the future.” (PS.P38) “I was quite fascinated with the way that we did the seminar... the 3D image that has been created, give us a lot of details, so we can have a deeper discussion on the artwork... this is really important because it facilitated our discussion later.” (3.2P4) “I definitely like the technology to support the exhibition, actually, because I think it’s quite easy and convenient for us to access different resources and also we did like four or five questions, we have to post our ideas or comments on the Padlet, and that actually helps to connect with people from different cultural backgrounds or different locations.” (6.2P4) “I felt there was a safe space to really share more about our personal perspectives about the artworks, and how they relate to health as a whole” (3.1P3).
Barriers to learning: pedagogical approach, learning resources, time factors, and virtual constraints	2% (1/49) somewhat disagreed that the online platform for interaction with the virtual museum objects was easy to use. 2% (1/49) somewhat disagreed that the preseminar online resources were useful preparation for the seminar experience.	“I think scale plays a really big role in how you emotionally respond to something, and that is something that’s lost through the digital gallery, unfortunately.” (1.1P2) “[It was] certainly difficult sometimes to [sic] looking at some of the pieces and trying to think of how it relates to health, and yeah, just bringing it back to the theme.” (6.2P1)
Novel and distinctive experiences: international, art-integrated learning	98% (48/49) agreed that engaging with virtual museum objects in the seminar allowed me to explore the health theme with peers in a way that was novel to them.	“But I think, generally, the virtual experience is really amazing, that you can actually get it all that work from different like different authors, different cultural backgrounds, that allow us to actually integrate it into the health care or into the humanity concept. So I think, yeah, it’s generally very, very unforgettable.” (6.1P4) “Creativity is such a, like, nourishing thing for the human spirit… and it’s something that in health care, you know, we don’t spend much time reflecting on it, because we’re so busy just getting the job done. So it’s wonderful to have these experiences where we share our thoughts about the world, and look at it from a different perspective, like using art as the, sort of, talking point, around which we can focus the discussion.” (2.1P3)

a COIL: collaborative online international learning.

## Discussion

### Principal Findings

A novel virtual health humanities approach for collaborative and international learning among health professional students, the HHGC, was developed. While the HHGC draws on established pedagogies (MB-HE, OBL, and COIL), its contribution to educational practice lies in their structured synthesis as a synchronous, virtual, cross-institutional seminar that uses shared, curated cultural objects as mediating tools. This integrated format creates an accessible “third-space” for intercultural dialogue that leverages material culture as a neutral focal point, supports small-group inquiry across institutions, and is designed for scalability and sustainable access. In this way, the HHGC offers a practicable model for embedding humanities-informed, intercultural learning into curricula where face-to-face exchanges or international placements are infeasible.

We found that the HHGC approach raised participants’ awareness of diverse cultural perspectives and provided stimulating opportunities to consider intercultural contexts through a humanities lens. We believe the perceived benefits of the seminar can be attributed to its unique design, which addresses gaps in traditional health education through a form of situated learning. By creating a supported and safe environment for dialogue mediated by virtual museum objects and artworks, the HHGC provided a psychologically safe environment where knowledge could be coconstructed, rather than merely transmitted [29]. As we had hypothesized, the HHGC structure allowed students to explore sensitive topics without the defensiveness that can arise in unmediated conversations, mitigating the risk of stereotyping. The objects served as a neutral focal point, enabling students to discover both surprising commonalities and profound cultural differences in a low-stakes context. As one student noted, this allowed for a more holistic overview. Several participants closely connected the HHGC’s discussions and focused analysis of artworks to their professional clinical experiences and appreciated the power of developing one’s ability to observe closely as they recognized these as transferable skills. This personal experience was coupled with the newly gained knowledge for comparing observations with group members of different cultural backgrounds and appreciating these differences, shedding bias, and establishing a more neutral yet holistic assessment. Some reflections written by participants offered suggestions for curriculum integration in broader health education contexts and outlined some potential applications in health care delivery. This will be valuable as we develop the methodology further.

We were particularly struck by how intellectually and creatively engaged the students typically were during the seminar, despite their perceived barriers such as unfamiliarity with OBL or the virtual learning context. As facilitators, we noticed how breakout room conversations were consistently active and productive, and how participants were confident to express new ideas about artwork interpretations, often drawing on their emotional responses to the artists’ themes or the appearance of the artworks. In the data, we found many instances where participants used adverbial intensifiers in their descriptions of the value or personal impact of the experience, such as “pretty eye opening,” “very surprising,” and “genuinely, really, shocked” (in a positive sense). We consider that this suggests that participants experienced moments of reflexive self-confrontation at points in the seminar, potentially signaling the beginning of greater awareness and humility that is foundational to authentic intercultural engagement [30].

Considering our experiences in delivering the HHGC, and the data from our study showing the students’ experiences, we posit that the methodology could be valuable in promoting cultural humility in students. Cultural humility, as originally described by Tervalon and Murray-García [30], is a lifelong process of self-evaluation and critique of one’s own cultural biases and limitations, oriented toward redressing power imbalances in the clinical encounter [31]. Bora et al [32] include cultural humility as one of the key elements when setting up cross-cultural dialogue in psychiatry training, along with open-ended inquiry, reflective practice, active listening, didactic education, and a collaborative approach. As a distinct element in a suite of approaches such as those described by Bora et al [32], the HHGC could serve as a stimulating humanities-based context to access diverse cultural perspectives. Our HHGC methodology centers ambiguity, interpretation, and highlights the validity of multiple perspectives. For these reasons, it may be a fruitful pedagogical tool in a larger approach that pushes back against the “hidden curriculum” of biomedicine, which often privileges certainty and a single, objective truth [31,33].

### Strengths and Limitations

We believe this study has provided evidence for the potential of our novel pedagogical approach to support participants’ development toward the important educational goal of increased intercultural competence and awareness. The curriculum, through being delivered in virtual learning environments, supports the goals of sustainable and equitable access to internationalizing experiences for learners in the health professions. However, our study of this pedagogy faced limitations. Importantly, the outcomes reported in this study reflect participants’ immediate, self-reported perceptions of learning following a single 2-hour seminar, rather than direct measures of demonstrated competence. These findings, therefore, indicate perceived changes in awareness, confidence, and analytic orientation, rather than objective or sustained changes in intercultural skills. Future work should assess demonstrated competence using validated performance-based measures and longitudinal follow-up to determine whether perceived gains translate into durable, observable changes in behavior and clinical practice. Further limitations include self-selection bias from the recruited cohort: the sample reflects students who were available and willing to participate in a humanities-oriented virtual seminar and may therefore be more favorably disposed to art-integrated learning and online learning than the broader student population. The geographic representation, including participants drawn from universities in Australia, Singapore, and Hong Kong, while offering some diversity, does not capture the full range of global health education contexts and cultures, while the recruitment of primarily English-fluent participants limits generalizability. These sampling constraints should be borne in mind when considering the transferability of results to other settings.

Finally, the application of the HHGC methodology into health education contexts has specific development and delivery factors, requiring expert facilitators who have the skills and inclination to collaborate across the disciplines of health professional education and museum-based tertiary education, as well as access to compelling digital art resources. Additional challenges for delivery into the curriculum in health education include complex scheduling for cohorts learning together across multiple international sites and time-zones, a characteristic common to COIL educational programming, but it is acutely the case in the very full curriculum common in health education contexts.

### Future Directions

We believe that the methodology of the HHGC has the potential to be translated into a range of health education contexts, by shaping the OBL context around a range of health themes, or broader topics such as sustainability in health care provision, or interprofessional collaboration and leadership. The HHGC methodology may be embedded into educational activities in curricula, either as short single-seminar interventions or as multiweek COIL projects. However, further research on the impact of these embedded experiences, on their longitudinal impact on students’ cultural awareness or development of cultural humility, and on how they might shape students’ interactions with their patients in clinical environments would be highly valuable to identify areas in which the methodology would be most effectively implemented and where it can be improved. Implementation research could also compare virtual versus in-person OBL experiences to clarify the trade-off between accessibility and embodied engagement with objects.

### Conclusions

The proposed HHGC methodology has the potential to be implemented in health professions learning, where there is an educational need to promote intercultural capabilities. This pilot study suggests that the HHGC could offer a sustainable, scalable, and replicable approach for educators as a resource to educate future health care professionals with the critical personal and intercultural awareness necessary to provide humane care in a globalized world.

## Abbreviations

COIL: collaborative online international learning
HHGC: Health Humanities Global Classroom
HKU: University of Hong Kong
MB-HE: museum-based health education
NUS: National University of Singapore
OBL: object-based learning
UoM: University of Melbourne
